# Supplementary figures and images for: An optimized approach to study nanoscale sarcomere structure utilizing super-resolution microscopy with nanobodies
Source: PLoS One. 2024 Apr 30;19(4):e0300348. doi: 10.1371/journal.pone.0300348 (PMC11060602; doi:10.1371/journal.pone.0300348)

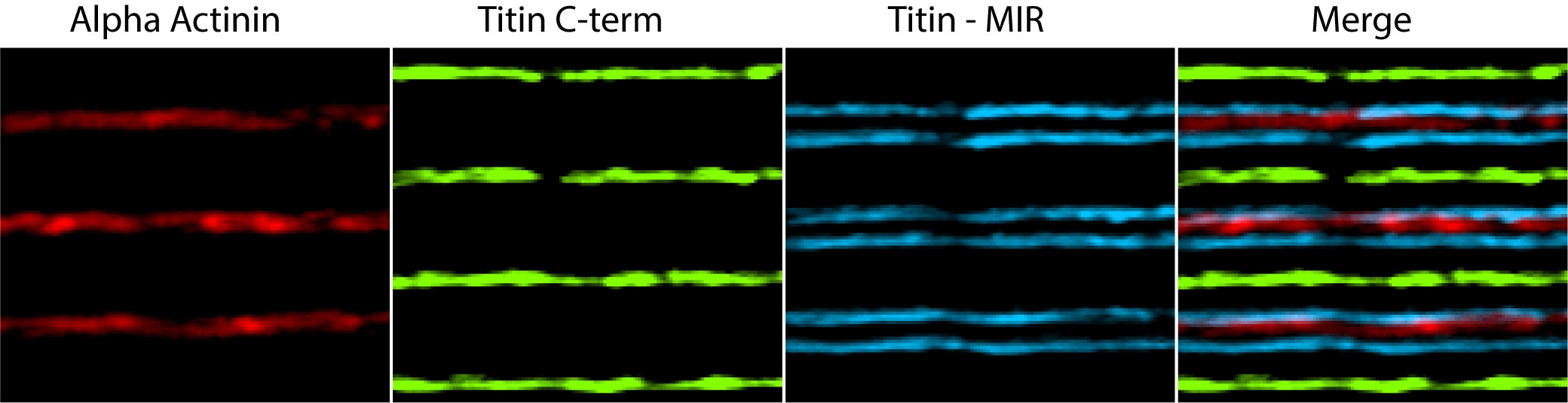

Supplement: S1 Fig — (TIF) [file pone.0300348.s001.tif]

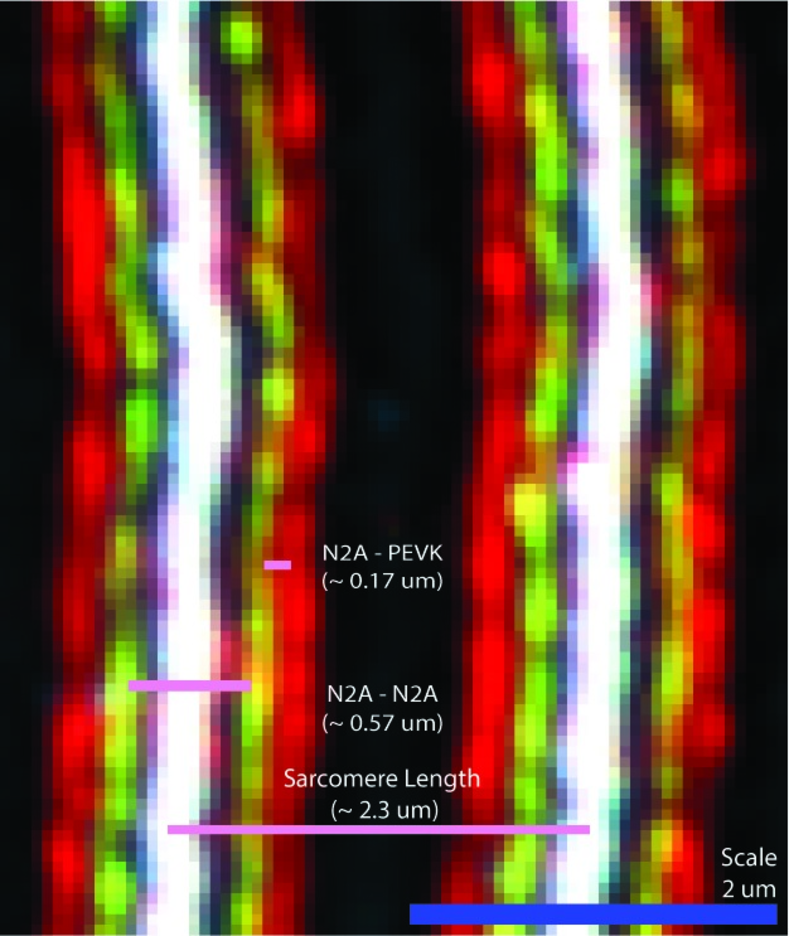

Supplement: S2 Fig — (TIF) [file pone.0300348.s002.tif]
